# The Long-Term Impact of the COVID-19 Pandemic on Loneliness in People Living With Disability and Visual Impairment

**DOI:** 10.3389/fpubh.2021.738304

**Published:** 2021-09-09

**Authors:** Nikki Heinze, Syeda F. Hussain, Claire L. Castle, Lauren R. Godier-McBard, Theofilos Kempapidis, Renata S. M. Gomes

**Affiliations:** ^1^Research and Innovation, Blind Veterans UK, London, United Kingdom; ^2^BRAVO VICTOR, Research, London, United Kingdom; ^3^Veterans and Families Institute for Military Social Research, Anglia Ruskin University, Chelmsford, United Kingdom; ^4^Northern Hub for Veterans and Military Families Research, Department of Nursing, Midwifery and Health, Faculty of Health and Life Sciences, Northumbria University, Newcastle upon Tyne, United Kingdom

**Keywords:** loneliness, UCLA, disability, visual impairment, sight loss, COVID-19

## Abstract

**Background:** There has been growing concern about the impact of restrictions put in place to contain the coronavirus pandemic on loneliness, particularly in individuals with disabilities. This study explored the longitudinal impact of the pandemic on loneliness in these individuals, with a focus on those living with visual impairment (VI).

**Methods:** An online survey was conducted in April-2020 and repeated in March 2021 to explore current life circumstances, health-related behaviours, sleep (Pittsburgh Sleep Quality Index) and social well-being, including state anxiety (State-Trait Anxiety Index) and loneliness (UCLA Loneliness scale). A convenience sample of 602 participants completed the first survey. Participants who agreed to be re-contacted were invited to take part in the follow-up survey.

**Results:** Data is presented for the 160 participants who completed both timepoints. At both timepoints, median loneliness was significantly higher in participants with disabilities and those with VI than in participants with no disabilities. While there was no significant change in loneliness in any of the three subgroups, participants with VI experienced the largest increase in median loneliness. Loneliness was associated with having a mental health condition and higher levels of state anxiety at both timepoints.

**Conclusions:** Individuals with disabilities such as VI experienced consistently higher levels of loneliness than those with no disabilities throughout the pandemic. While loneliness remained relatively stable in individuals with no disabilities, it increased, albeit to a non-significant level, in those with disabilities and particularly those with VI. Interventions designed to alleviate loneliness may benefit from addressing state anxiety.

## Introduction

In response to the COVID-19 pandemic, governments around the world introduced a range of measures to contain the spread of the virus, such as mask-wearing indoors and/or outdoors, curfews and national lockdowns. The timing and strictness of the measures implemented varied between countries. The United Kingdom (UK) government, for instance, announced national lockdowns on 23 March 2020, and again on 5 November 2020 and 6 January 2021, requiring schools and non-essential retail to close, and people to stay indoors except for essential purposes, such as food shopping, medical appointments, outdoor exercise and work where working from home was not possible. Lockdowns, and other restrictions on social contact such as social distancing and the introduction of working-from-home policies, have acted as physical barriers to interactions with family, friends, colleagues, and communities, and there has been concern about the impact of these restrictions on experiences of social isolation and loneliness (1, 2). Research carried out during the early months of the pandemic in the UK found that between 27 and 35.9% of study participants felt lonely at least sometimes (3, 4). Figures from the Office for National Statistics (5) further showed that 5.0% of the UK adult population felt lonely often or always between 3 April and 3 May 2020, increasing to 7.2% between October 2020 and February 2021. A significant increase in loneliness over the first 6 months of the pandemic was also observed in the US (6), although others suggest that loneliness has remained relatively stable throughout the pandemic (7, 8). Dean et al. (9) reported statistically significant cross-cultural differences with higher levels of loneliness found in Hong Kong (*M* = 49.7) compared to South Korea (*M* = 43.3), the US (*M* = 41.2) and France (*M* = 35.8). Higher loneliness during the pandemic has been associated with being female, younger, from an ethnic minority background, having a lower income, living alone, living in cities or towns, being separated or divorced, and having poor sleep (3, 4, 8, 10). The relationship between sleep quality and loneliness is thought to be reciprocal, with both impacting on the other (11).

There has also been concern about the impact of the pandemic on people with disabilities (12, 13). Research suggests that loneliness was higher among vulnerable populations such as those living with chronic illness (7) and pre-existing physical and mental health conditions (8), although, as in the general population, loneliness appears to have remained relatively stable among these groups (7, 8). Even before the pandemic, individuals living with a disability were at increased risk of loneliness, self-isolation, and mental health difficulties (14–16). Emerson et al. (15) found that working-age adults with disability in England were 51% more likely to report social isolation, and around four times more likely to report loneliness than those with no disability. Loneliness was particularly prevalent in younger, economically inactive adults with disability who lived alone and had little access to community amenities such as local shops and parks (15). Research has also highlighted the impacts of different types of disability on loneliness. Macdonald et al. (14) found that loneliness and isolation were particularly high among those with learning difficulties or disabilities, and mental health issues. Similarly, Mithen et al. (17) found that of those with a disability, individuals with intellectual and psychological disabilities had the lowest levels of social capital, including social contact. Visual impairment (VI) has often been associated with greater incidence of loneliness, particularly in older adults (18–21) and those with comorbid conditions (16, 22). This may partly reflect the barriers to participation in social, leisure (23), and occupational (24–26) contexts that people with VI often face, including inaccessible environments and transport (27, 28). However, Macdonald et al. (14) reported lower prevalence of loneliness and isolation in individuals with physical and sensory impairment compared to other disabilities, and Wahl et al. (29) found no significant difference in loneliness between older adults with VI and those with no sensory loss, hearing impairment, or dual sensory loss. Despite this, a UK study by Ting et al. (30) suggests that individuals with moderate to severe VI were three times more likely to report increased levels of loneliness than those with mild or no VI during the pandemic. Loneliness may be a particular concern for visually impaired individuals with Charles Bonnet Syndrome, for whom increased loneliness during the pandemic has been associated with more troublesome hallucinations, and greater difficulty distinguishing between hallucinations and reality (31).

Existing research has consistently highlighted an association between loneliness and negative health outcomes, including early mortality (32, 33), coronary heart disease and stroke (34), and poor sleep (35–37). Loneliness has also been linked to mental health difficulties (38–40) and low levels of well-being in individuals with disability (15). Considering the association of disability and increased physical and mental health challenges (41, 42), and the potential impact of disability on loneliness discussed above, it is essential to understand the impact that the pandemic has had on experiences of loneliness amongst individuals living with a disability. The current paper explores the longitudinal impact of the COVID-19 pandemic on loneliness in people living with disability. Given mixed evidence of loneliness in individuals with VI prior to the pandemic, and reports of higher levels of loneliness in this group during the pandemic, the paper will further focus on the longitudinal impact on loneliness in individuals with VI.

## Materials and Methods

Data was collected at two timepoints (T1 and T2) to assess the long-term impact of the COVID-19 pandemic on individuals with disability.

### Materials

An online survey was developed by the Research and Innovation team at Blind Veterans UK, a charity providing support to visually impaired British veterans, in collaboration with the University of Oxford. Ethical approval for this study was not required, as advised by the Medical Sciences Interdivisional Research Ethics Committee at the University of Oxford. Following participant information, consent and demographics, the questionnaire consisted of four sections addressing current life circumstances (e.g. employment status, living situation), general health (e.g. self-isolation and disability status) including health-related habits and behaviours (e.g. alcohol consumption), sleep quality and social well-being (loneliness, anxiety). The questionnaire was amended for T2 to improve data quality and reduce participant burden (see details below). A small number of participants across both timepoints (*n* < 10) contacted the research team to conduct the survey over the phone.

#### Disability Status

At T1, participants were first asked if they had a disability. This was followed by a question which instructed them to select all disabilities that applied to them from a list of 16 conditions which included VI or blindness, acquired brain injury, medical conditions such as diabetes, arthritis or epilepsy, disability affecting mobility, mental health issues, and learning difficulties, amongst others. At T2, participants were asked if they considered themselves to have a disability and the list of conditions was turned into a grid which required participants to select “Yes,” “No,” or “Prefer not to say” for each condition.

#### Sleep Quality

Sleep quality over the last month was assessed using the Pittsburgh Sleep Quality Index (PSQI) (43). The PSQI is a self-report measure consisting of 19 items which are used to derive seven component scores (subjective sleep quality, sleep latency, sleep duration, sleep efficiency, sleep disturbance, use of sleep medication and daytime dysfunction). The component scores are summed to derive a global PSQI score ranging from 0 to 21, with higher scores indicating worse sleep quality.

#### State Anxiety

Anxiety was measured using the 20-item state anxiety subscale of the State Trait Anxiety Index (STAI-S) (44). The STAI-S scale consists of ten positively worded statements and ten negatively worded statements which together assess current (state) anxiety, rather than trait anxiety. Respondents are instructed to indicate how they are feeling “right now” on a scale of 1 (Not at all) to 4 (Very much). Positively worded items are reverse-scored and all scale responses are summed to derive a subscale score ranging from 20 to 80, with higher scores indicative of greater anxiety.

#### Loneliness

Loneliness was assessed using version 3 of the UCLA Loneliness scale (45). It consists of 20 items which measure subjective feelings of loneliness and social isolation. The scale has been employed across multiple populations and large-scale studies, including elderly populations (45, 46), and those with physical disabilities or sensory loss (47–49). Respondents are instructed to indicate how often they feel lonely or socially isolated on a scale of 1 (Never) to 4 (Often). A loneliness score is derived by summing scale responses resulting in a score ranging from 20 to 80. Simple mean imputation was used in cases where one or two responses were missing. The steps were as follows: the mean for the item scores provided was calculated; this value was then inputted as the mean of the of the value that was missing; the final score was calculated using continuous scoring so this was a summation of all input values. The derived score was rounded to an integer.

### Recruitment

Data collection for T1 took place between 1 April 2020 and 15 May 2020. A convenience sample of participants was recruited via email, with a link to the online survey, through the researchers' personal and professional networks, social media platforms, and professional forums. Data collection for T2 took place between 8 March 2021 and 28 March 2021. Participants were invited to take part if they had consented to being re-contacted and provided a valid email address in the previous survey.

### Procedure

At the start of the survey, participants were provided with information about the study and their rights as participants, before being asked to consent to taking part. Participants were able to select if they wanted to skip any of the four main sections. As a result, the number of responses for each section varied. Participants who were happy to be re-contacted for follow-up surveys were asked to provide an email address or contact telephone number at the end of T1.

### Analysis

The current paper set out to explore changes in loneliness in individuals with disability, and those with VI, compared to those with no disabilities. Data is presented for participants who completed T1 and T2. Duplicates and cases without valid responses for T1 and T2 were removed from the dataset prior to analysis. “Prefer not to say” was treated as a missing value in the analysis.

Subgroup analysis was carried out by disability status (participants reporting one or more disabilities, including VI, vs. participants with no disabilities) and VI (participants who reported having VI vs. participants with no disabilities). It should be noted that participants with VI are also included in the group of participants with one or more disabilities. It was also not possible to control for comorbid disabilities in participants with VI due to the small number of participants who reported having VI only.

A typographical error meant that one of STAI-S scale items (Q4) was presented incorrectly in T1. This was corrected in T2. A revised anxiety score was calculated for both surveys which excludes the incorrect item. The revised scores were used in analysis to enable comparison.

Loneliness scores were not normally distributed, as assessed by Shapiro-Wilk's test (*p* < 0.05). As a result, non-parametric tests using medians were performed. First, between-group differences in loneliness in people with disabilities and those with VI compared to those with no disabilities were assessed at both timepoints using Mann-Whitney U tests. Second, changes in loneliness within each subgroup were assessed using Wilcoxon signed rank tests and sign test. Third, a hierarchical regression was performed to identify factors associated with loneliness at T1 and to assess the role of VI when controlling for other factors. The regression was repeated at T2 to identify stable factors.

## Results

### Participant Characteristics

Table 1 provides an overview of participant characteristics. After removing duplicates, a total of 602 participants completed T1, 329 of whom were invited to take part in T2 with 163 yielding responses (49.5%). After removing two cases who had indicated that they did not wish to take part in T2 and one case who had submitted T1 twice with different answers, a total of 160 completed T1 and T2. The majority of these participants were female, white, aged 46–55, resident in the UK, in paid employment, living with others e.g. family, had been self-isolating for 2–4 weeks in T1 and were not self-isolating in T2. Overall, sleep was poor in this sample. The mean PSQI global sleep score at T1 was 7.01 (*SD* = 4.31), with 55.6% of participants being categorised as poor sleepers (a PSQI global score of >5). At T2, the mean PSQI global sleep score was 8.02 (*SD* = 4.59), with 59.4% of participants being categorised as poor sleepers. Mean anxiety using the revised score was 38.89 (*SD* = 14.22) at T1 and 38.08 (*SD* = 14.27) at T2.

**Table 1:** Sample characteristics.

		**%** (***n***)	**T1 %** (***n***)	**T2 %** (***n***)
Gender	Female	52.2 (83)		
	Male	47.8 (76)		
Ethnicity	Asian	1.3 (2)		
	Black/African/Caribbean	0.6 (1)		
	Hispanic/Latino/Spanish origin	1.9 (3)		
	Mixed/multiple ethnic groups	1.3 (2)		
	White/Other White	95.0 (151)		
Country of residence^a^	Canada	0.6 (1)		
	France	1.3 (2)		
	Germany	1.9 (3)		
	Greece	0.6 (1)		
	Malta	5.6 (9)		
	Portugal	3.1 (5)		
	Thailand	0.6 (1)		
	UK	76.9 (123)		
	USA	9.4 (15)		
Age	18–25		1.9 (3)	1.9 (3)
	26–35		13.9 (22)	11.4 (18)
	36–45		13.9 (22)	17.7 (27)
	46–55		34.8 (55)	31.0 (49)
	56–65		25.9 (41)	24.1 (38)
	66–75		6.3 (10)	10.8 (17)
	76–85		3.2 (5)	3.2 (5)
	86+		–	–
Employment status	In paid employment		73.5 (111)	69.6 (110)
	I am employed but furloughed		–	1.3 (2)
	Retired		16.6 (25)	17.7 (28)
	Unemployed and not looking for work		7.9 (12)	9.5 (15)
	Unemployed but looking for work		2.0 (3)	1.9 (3)
Time spent self-isolating	I'm not self-isolating		24.5 (39)	70.9 (112)
	0–2 weeks		5.7 (9)	0.6 (1)
	2–4 weeks		42.1 (67)	-
	4–8 weeks		27.0 (43)	0.6 (1)
	8–12 weeks		1.3 (2)	1.3 (2)
	Over 12 weeks (T1)/3–4 months (T2)		0.6 (1)	0.6 (1)
	4–5 months (T2 only)		–	1.3 (2)
	Over 6 months (T2 only)		–	24.7 (39)
Living status	I live on my own		23.9 (38)	23.9 (38)
	I live with others		76.1 (121)	76.1 (121)
Disability	No disability	66.5 (105)		
	One or more disabilities	33.5 (53)		
VI or blindness	VI not reported	76.9 (123)		
	VI reported	23.1 (37)		

a *The country of residence question was not repeated in T2*.

#### Disability and VI

Around two thirds reported no disabilities, while a third of respondents reported having one or more disabilities; with a maximum of eight distinct types of disabilities being reported by one participant. The most commonly reported type of disability was VI or blindness (23.1%), followed by disability affecting mobility (16.3%), mental health conditions (13.1%), medical conditions such as epilepsy, asthma and diabetes (12.5%), and hearing impairment or deafness (11.3%). Of the 37 participants who had a VI, nine reported having a VI alone and 28 participants reported a comorbid condition, 48.6% had a disability affecting mobility and 43.2% a hearing impairment. Due to the small T2 sample size it was not possible to control for other disabilities in the “VI” group during analysis.

### Between-Group Comparison: Loneliness by Subgroup at T1 and T2

**Figure 1:**
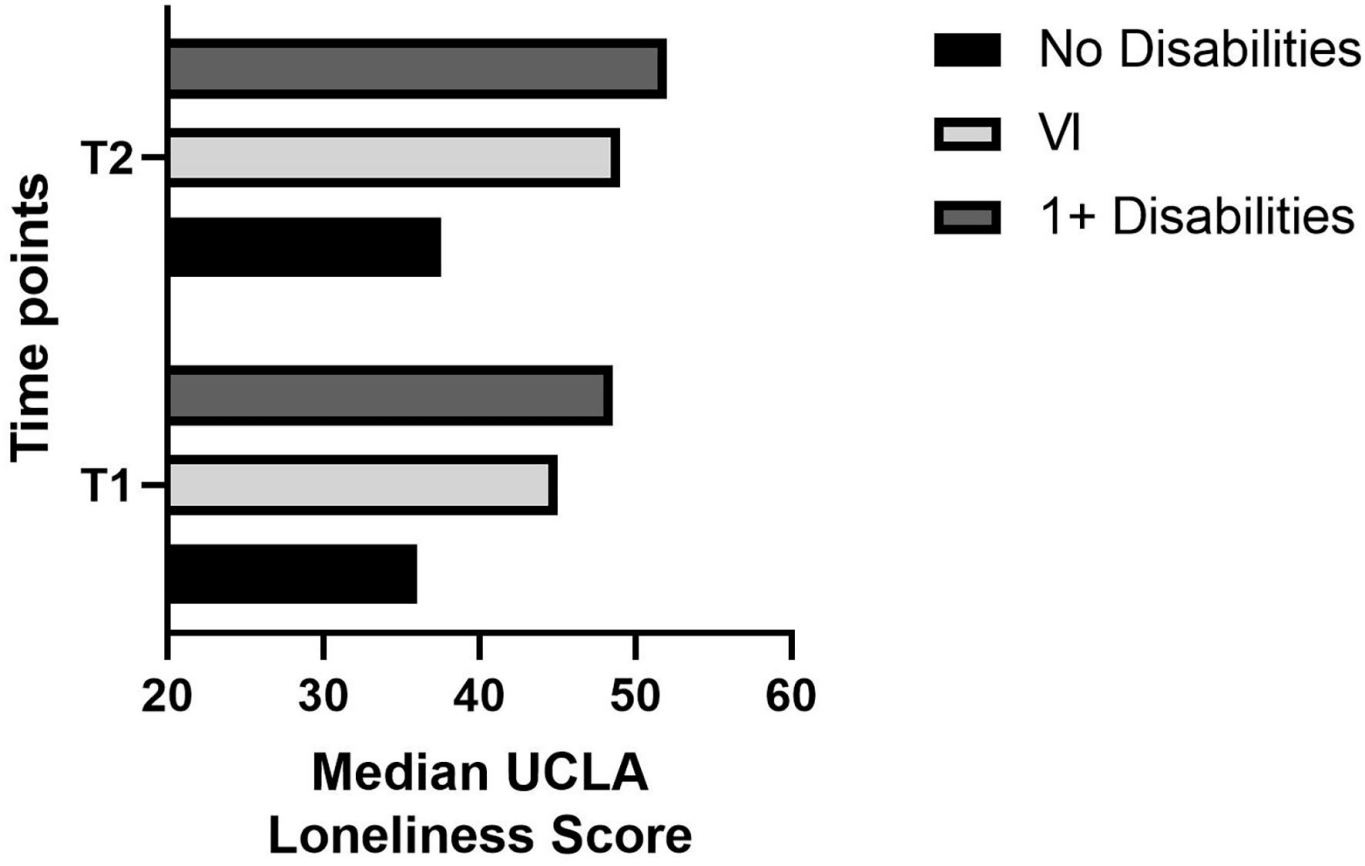
Median UCLA loneliness score by subgroup at T1 and T2.

Means, standard deviations, medians, interquartile ranges, and number of valid cases for loneliness by subgroup are reported in Table 2. Mean loneliness was highest in participants with disabilities and lowest in those with no disabilities at both timepoints. As seen in Figure 1, median loneliness was significantly higher in participants with one or more disabilities, *U* = 3,523, *p* < 0.001, and in participants with VI, *U* = 2,242.5, *p* < 0.05, than in participants with no disabilities.

**Table 2:** Descriptive statistics for UCLA loneliness by subgroup at T1 and T2.

	**T1**			**T2**		
	**No disabilities**	**1+ disabilities**	**VI present**	**No disabilities**	**1+ disabilities**	**VI present**
*n*	103	50	35	104	51	35
*M (SD)*	37.21 (11.03)	46.92 (15.58)	44.20 (15.87)	38.78 (12.67)	48.63 (15.52)	46.40 (15.95)
*Mdn* (*IQR*)	36.0 (15)	48.5 (28)	45.0 (30)	37.5 (20)	52.0 (28)	49.0 (26)

*n, number of valid cases; M, mean; SD, standard deviation; Mdn, median; IQR, interquartile range*.

At T2, mean loneliness continued to be higher in participants with disabilities and VI than in those with no disabilities. As seen in Figure 1, median loneliness was, again, significantly higher in participants with disabilities, *U* = 3,660, *p* < 0.001, and in participants with VI, *U* = 2,345.5, *p* < 0.05, than in participants with no disabilities.

### Within-Group Comparison: Changes in Loneliness Over Time

**Figure 2:**
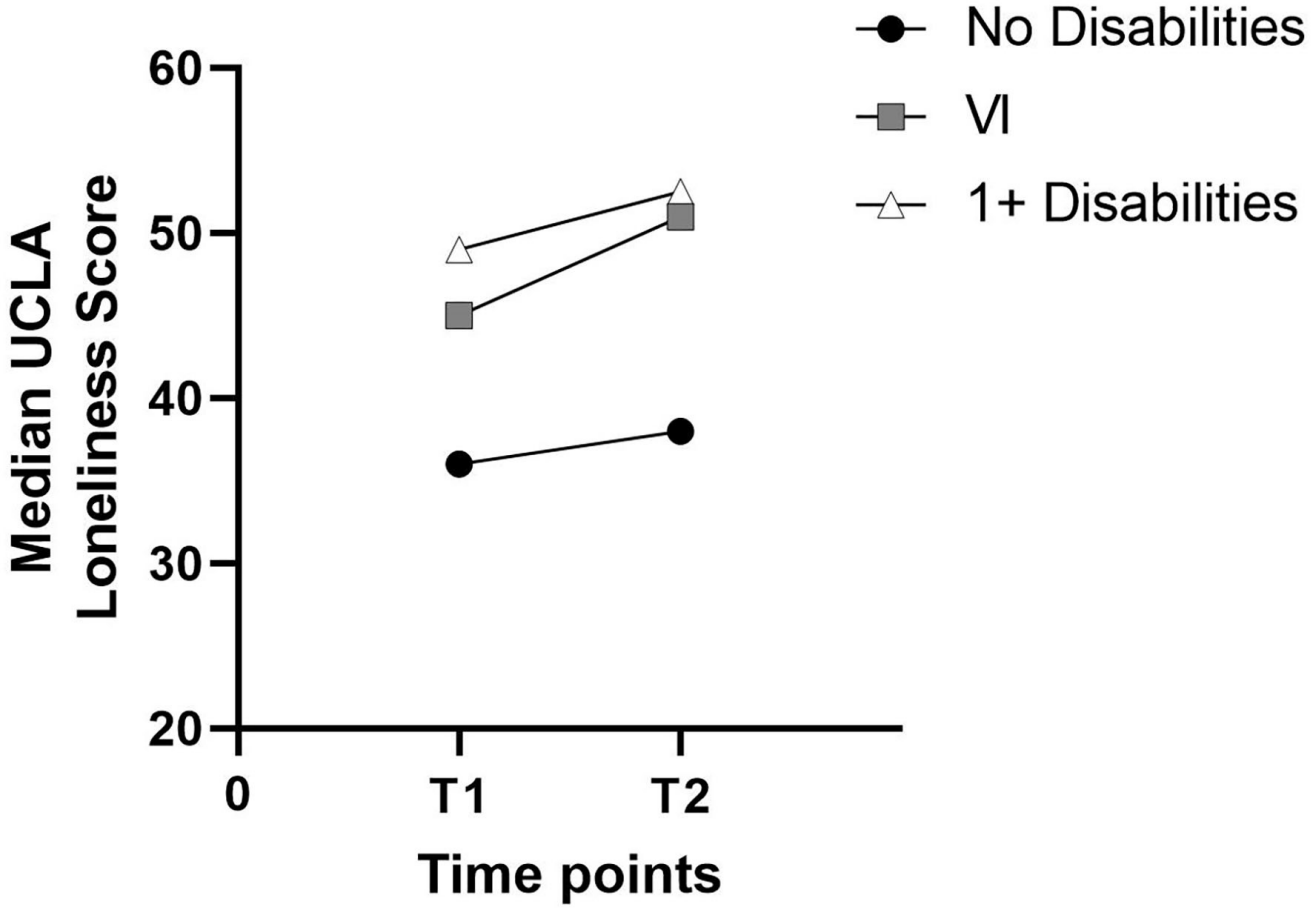
Change in median UCLA loneliness between T1 and T2 by subgroup.

Loneliness scores for both surveys were available for 103 participants without disabilities, 48 with disabilities, and 33 participants with VI. Although mean and median loneliness increased in all three groups (see Figure 2), median loneliness did not differ significantly between the two surveys for participants without disabilities, *T* = 2,941, *p* = 0.068, nor for participants with disabilities, *T* = 554, *p* = 0.883. The distribution of the difference between T2 and T1 scores was not symmetrical for participants with VI so a sign test was carried out. This showed that, although higher than for the other two groups, the median increase in loneliness in participants with VI was also not statistically significant, *z* = −0.530, *p* = 0.596.

### Factors Predicting Loneliness

Hierarchical regressions were run for both surveys to determine the relationship between VI and loneliness when controlling for age and gender in the first step, anxiety (revised), sleep quality, time spent self-isolating, and living alone in step 2, and the more common types of comorbid disabilities in participants with VI such as disability affecting mobility, hearing impairment, medical conditions (e.g. asthma, diabetes, epilepsy) and mental health conditions, in step 3. Table 3 provides full details of the four regression models produced for T1. The full model of age, gender, anxiety, sleep quality, self-isolation, living situation, hearing impairment, disability affecting mobility, medical conditions, mental health issues and VI (Model 4) was statistically significant, *F*_(11,131)_ = 15.28, *p* < 0.001; adjusted *R*^2^ = 0.525. The addition of anxiety, sleep quality, self-isolation and alcohol consumption in Model 2 explained an additional 46.4% of the variance in loneliness above and beyond age and gender, *F*_(6,136)_ = 25.51, *p* < 0.001, adjusted *R*^2^ = 0.509. The addition of hearing impairment, disability affecting mobility, medical conditions, and mental health issues in Model 3 accounted for an extra 3.0% of the variance in loneliness, however this increase was not statistically significant (*p* = 0.067), adjusted *R*^2^ = 0.526. The addition of VI in the final model did not improve the ability of the model to explain loneliness. The variables with a significant contribution to explaining loneliness in the final model were being male, higher levels of anxiety, living alone, and having a mental health issue.

**Table 3:** Hierarchical multiple regression for UCLA loneliness at T1.

**T1**	**UCLA loneliness score**							
	**Model 1**		**Model 2**		**Model 3**		**Model 4**	
**Variable**	***B***	**β**	***B***	**β**	***B***	**β**	***B***	**β**
Constant	45.038***		16.595**		19.154***		19.167***	
Age	−0.155	−0.149	0.031	0.029	0.019	0.019	0.025	0.024
Sex	6.986**	0.262	7.544***	0.283	6.829***	0.256	7.280***	0.273
Anxiety (revised)			0.539***	0.559	0.532***	0.552	0.530***	0.550
Sleep quality			0.403	0.131	0.059	0.019	0.064	0.021
Self-isolation			0.220	0.018	0.113	0.009	0.077	0.006
Living situation			−7.127***	−0.230	−6.800***	−0.219	–7.046***	−0.227
Hearing impairment					−2.260	−0.047	−1.377	−0.029
Disability affecting mobility					2.888	0.077	3.502	0.093
Medical condition					−2.475	−0.059	−2.228	−0.053
Mental health issues					7.729*	0.197	7.683*	0.196
VI							−2.113	−0.065
*R^2^*	0.066		0.530		0.560		0.562	
*F*	4.95**		25.51***		16.77***		15.28***	
*ΔR^2^*	0.066		0.464		0.030		0.002	
*ΔF*	4.95**		33.50***		2.25		0.69	

*N = 143. B = unstandardized regression coefficient; β = standardised coefficient; R^2^ = coefficient of determination; ΔR^2^ = R^2^ change; ΔF = F change*.

* *p < 0.05*.

** *p < 0.01*.

*** *p < 0.001. Bold values in Model 4 denote significant predictors*.

Table 4 shows the full details for the four models produced for T2. The full model of age, gender, anxiety, sleep quality, self-isolation, living situation, hearing impairment, disability affecting mobility, medical conditions such as asthma, diabetes, epilepsy, mental health issues and VI (Model 4) was statistically significant and explained 58.4% of the variance in loneliness, *F*_(11,132)_ = 16.83, *p* < 0.001, adjusted *R*^2^ = 0.549. The addition of anxiety, sleep quality, self-isolation and living status in Model 2 explained an additional 46.1% of the variance in loneliness above and beyond age and gender, *F*_(6,137)_ = 26.41, *p* < 0.001, adjusted *R*^2^ = 0.516. The addition of hearing impairment, disability affecting mobility, medical conditions, and mental health issues in Model 3 accounted for an extra 4.2% of the variance in loneliness, *F*_(10,133)_ = 18.24, *p* < 0.001, adjusted *R*^2^ = 0.547. The addition of VI in the final Model 4 accounted for an extra 0.6% of the variance in loneliness; this increase was not statistically significant (*p* = 0.188). As for T1, higher levels of state anxiety and having a mental health issue were significant predictors of loneliness. Unlike T1, sex and living status did not contribute to predicting loneliness at T2 but not having a chronic medical condition did.

**Table 4:** Hierarchical multiple regression for UCLA loneliness at T2.

**T2**	**UCLA loneliness score**							
	**Model 1**		**Model 2**		**Model 3**		**Model 4**	
**Variable**	***B***	**β**	***B***	**β**	***B***	**β**	***B***	**β**
Constant	50.048***		15.786**		16.305**		15.987**	
Age	−0.232*	−0.213	−0.062	−0.057	−0.032	−0.029	−0.035	−0.032
Sex	7.696**	0.269	4.173*	0.146	4.170*	0.146	3.500	0.122
Anxiety (revised)			0.605***	0.594	0.583***	0.572	0.582***	0.571
Sleep quality			0.517*	0.165	0.352	0.112	0.395	0.126
Self-isolation			−0.081	−0.020	0.048	0.012	−0.007	−0.002
Living situation			0.380	0.011	−0.094	−0.003	0.193	0.006
Hearing impairment					−0.601	−0.013	−2.244	−0.048
Disability affecting mobility					1.339	0.034	0.240	0.006
Medical condition					−8.907**	−0.195	–9.314**	−0.204
Mental health issues					7.870*	0.186	8.006*	0.189
VI							3.579	0.104
*R^2^*	0.076		0.536		0.578		0.584	
*F*	5.77**		26.41***		18.24***		16.83***	
*ΔR^2^*	0.076		0.461		0.042		0.006	
*ΔF*	5.77**		34.03***		3.31*		1.75	

*N = 144. B = unstandardized regression coefficient; β = standardised coefficient; R^2^ = coefficient of determination; ΔR^2^ = R^2^ change; ΔF = F change*.

* *p < 0.05*.

** *p < 0.01*.

*** *p < 0.001. Bold values in Model 4 denote significant predictors*.

## Discussion

This study provides a preliminary assessment of loneliness in people living with disabilities, with a focus on those with VI, over the course of the COVID-19 pandemic. The study found no statistically significant deterioration in loneliness in any of the three subgroups assessed between April 2020 and May 2021, despite a slight increase in mean and median loneliness in participants with disabilities and VI. This reflects previous findings which suggest that, despite the restrictions on social contact, levels of loneliness have remained relatively stable during the pandemic (7, 8). As with existing research carried out during the pandemic (8, 30), loneliness was significantly higher in those with disabilities, including those with VI, than in individuals with no disabilities at both timepoints. Indeed, the mean loneliness scores for participants with no disabilities (37.21 ± 11.03 and 38.78 ± 12.67) are comparable to those reported for individuals with no sensory loss prior to the pandemic (38.49 ± 11.47) (29). It is possible that this group did not experience any marked negative impact of the pandemic on loneliness, or that loneliness had reverted back to pre-pandemic levels by the time of this study.

In contrast, mean loneliness in participants with VI was higher at both timepoints (44.20 ± 15.87 and 46.40 ± 15.95) than the UCLA mean previously reported for visually impaired individuals, both during (39.19 ± 14.04) (50) and prior (39.50 ± 12.85) to the pandemic (29). This may be due to the prevalence of comorbid conditions among participants with VI. However, mean loneliness in the VI group was also higher than the pre-pandemic score reported by Wahl et al. (29) for individuals with comorbid hearing and sight loss (41.11 ± 11.61). Research has proposed a U-shaped trajectory of loneliness across the adult age span, with higher levels of loneliness reported in younger adults and again in the oldest (51–54). This suggests that higher loneliness scores should be expected in Wahl et al.'s older sample (*M* = 83 years) compared to the current study. In addition, whilst not statistically significant, the VI group experienced the highest increase in median loneliness. Subjective physical health and limitations on daily activities are associated with loneliness in young and middle-aged adults, but not older adults in the UK (54). It is possible that, whilst neither age nor VI were associated with loneliness in the current study, limitations on social contact and daily functioning arising from VI may help to explain the comparatively higher levels of loneliness observed for participants with VI in the current study.

A number of factors predicted loneliness at one timepoint only, including being male and living alone at T1, and not having a chronic medical condition at T2. It is unclear why not having a medical condition was found to predict loneliness at T2 given that having a chronic health condition has previously been linked with loneliness (7, 8). Early findings from the UK suggest that in the majority of cases (67%), the care provided to those categorised as clinically extremely vulnerable, who were advised to shield, remained largely unaffected by the pandemic (55). Thus, individuals with a medical condition may have experienced a greater level of health and social support than others during this time. Self-isolation itself did not contribute to loneliness in either model in the current study, which may reflect the distinction made between physical isolation and feelings of isolation in the literature surrounding loneliness during the pandemic (56). Lewis (57) found that in-person interactions during this time had no impact on feelings of loneliness, whilst virtual contact was negatively associated with loneliness. Virtual contact maintained with support services and loved ones may have mediated the impacts of self-isolation on loneliness in the current sample. In contrast, having mental health difficulties was associated with loneliness in the current study, lending support to similar findings reported elsewhere (3, 8, 10). Reflecting this, higher levels of state anxiety were found to be significant predictors of loneliness across both timepoints. Anxiety relating to increased health, social, and financial concerns during the pandemic may have resulted in stricter self-imposed social distancing measures which increased feelings of loneliness (58). However, causality cannot be inferred. Indeed, mental health difficulties have also been identified as a potential consequence of loneliness (33, 37–40) and loneliness predicted state anxiety at both timepoints in the same sample (Heinze et al. 2021, under review), suggesting that the relationship between loneliness and mental health may be reciprocal. Individuals with disabilities may be particularly affected by concerns about their health if social and medical care is no longer accessible, or perceived as accessible, due to COVID-19 restrictions (13). Providing support in managing state anxiety, as well as adequate practical advice and support regarding health and social care needs during the pandemic, appear to be essential steps in reducing its potential impact on loneliness in individuals both with and without a disability.

### Limitations and Future Directions

Whilst the current study offers insight into the experiences of loneliness in people living with disabilities, and factors associated with loneliness during the pandemic, a number of factors limit the generalisability of findings. Firstly, the study relied on a convenience sample recruited through professional networks and the membership of Blind Veterans UK (BVUK). As a result, it is difficult to extrapolate findings to the wider population. Members of BVUK may receive support and services relating to well-being, which may have impacted on loneliness in the sample. The current study did not ask about any pre-existing issues with, or support received for, loneliness. Secondly, the number of participants who completed T2 is considerably lower than for T1. Although there were no statistically significant differences between responders and non-responders in terms of sex, ethnicity, disability and employment status and living situation, those who were invited to but did not complete T2 may have had a different experience relating to loneliness. In addition, a greater proportion of non-responders were living outside the UK. These differences may have driven their non-response. Cross-cultural differences and variations in the messaging and measures introduced globally may have further impacted experiences of loneliness. Due to the small number of participants residing in countries outside the UK, comparative analysis was not possible. Future research will need to assess if the findings presented here for changes in loneliness hold in a larger sample of people with VI and disability, and the impact of cross-cultural differences on experiences of loneliness in these groups.

Another limitation of this research was the incorrect listing of STAI-S item 4 in T1. This meant that a revised score excluding the item had to be calculated for both surveys to enable comparison. The revised score has not undergone validation and the exclusion of item 4 may have impacted on findings relating to state anxiety and its relationship to loneliness. Additionally, the study compared individuals with and without disability but did not account for the comorbidity. For instance, this paper explored the experiences of individuals with VI but, due to small sample sizes, it was not possible to control for additional types of disabilities. Further research exploring if, and how, different types of disability, including physical, sensory, cognitive impairment, and mental health difficulties, and having one or multiple comorbidities, may have impacted on experiences of health and well-being during the pandemic is needed. Such exploration would offer a greater understanding of the long-term public health implications of the pandemic. In addition, the type and frequency of contact maintained by this sample during the pandemic was not explored in this study. Future research may need to explore the role of virtual and sporadic face-to-face contact in feelings of loneliness in self-isolating adults with disability.

### Conclusions

The current paper provides a preliminary assessment of loneliness in people living with disability with a focus on those living with sight loss. Loneliness remained relatively stable during the pandemic but was consistently higher in individuals with disabilities such as VI than in individuals with no disabilities. The highest increase in loneliness was observed in individuals with VI. Having a mental health condition and state anxiety were found to be stable predictors of loneliness at both timepoints. This suggests that any interventions designed to support individuals experiencing loneliness during the pandemic may benefit from targeting state anxiety.

## Data Availability Statement

The datasets presented in this article are not readily available because participants were not asked if they consented for their data to be shared outside of the research teams involved in this study. Requests to access the datasets should be directed to Renata S. M. Gomes, renata.gomes@blindveterans.org.uk.

## Ethics Statement

Ethical review and approval was not required for the study on human participants in accordance with the local legislation and institutional requirements. The patients/participants provided their written informed consent to participate in this study.

## Author Contributions

NH designed and performed the analysis and wrote the paper. SH wrote the paper and edited the paper. CC designed the survey, wrote the paper and edited the paper. LG-M consulted on data analysis and edited the paper. TK designed the survey, produced graphics for the paper and edited the paper. RG designed the survey and edited the paper. All authors contributed to the article and approved the submitted version.

## Funding

This study was funded by Blind Veterans UK.

## Conflict of Interest

The authors declare that the research was conducted in the absence of any commercial or financial relationships that could be construed as a potential conflict of interest.

## Publisher's Note

All claims expressed in this article are solely those of the authors and do not necessarily represent those of their affiliated organizations, or those of the publisher, the editors and the reviewers. Any product that may be evaluated in this article, or claim that may be made by its manufacturer, is not guaranteed or endorsed by the publisher.

## Abbreviations

T1: Timepoint 1 (survey 1)
T2: Timepoint 2 (survey 2)
VI: Visual impairment
PSQI: Pittsburgh Sleep Quality Index
STAI-S: State anxiety subscale of the State-Trait Anxiety Index.
